# *Clostridioides difficile* in national food surveillance, Slovenia, 2015 to 2017

**DOI:** 10.2807/1560-7917.ES.2020.25.16.1900479

**Published:** 2020-04-23

**Authors:** Valerija Tkalec, Urska Jamnikar-Ciglenecki, Maja Rupnik, Stanka Vadnjal, Katja Zelenik, Majda Biasizzo

**Affiliations:** 1 National Laboratory for Health, Environment and Food, Maribor, Slovenia; 2 Faculty of Medicine, University of Maribor, Maribor, Slovenia; 3 Institute of Food Safety, Feed and Environment, Veterinary Faculty, University of Ljubljana, Ljubljana, Slovenia

**Keywords:** Clostridium difficile, Clostridioides difficile, foodborne, food surveillance, vegetables, meat, reservoir

## Abstract

**Background:**

*Clostridioides difficile* is an important human and animal intestinal pathogen. Because of increasing indications of an association between *C. difficile* and food, in 2015, the Administration of the Republic of Slovenia for Food Safety, Veterinary Sector and Plant Protection (UVHVVR) included *C. difficile* in its national food surveillance.

**Aim:**

We aim to report the results and experience with a nationwide and long-term testing of food for *C. difficile* as a part of a regular national food surveillance programme.

**Methods:**

Retail minced meat and meat preparations (beef, pork and poultry) were sampled within a three-year period, 2015 to 2017. Selected raw retail vegetables, leaf salads and root vegetables, and ready-to-eat salads were only sampled during 2016 and 2017. Seafood was only sampled in 2017.

**Results:**

Altogether, 434 samples were tested, with 12 of 336 (3.6%) meat samples and 6 of 98 (6.1%) raw vegetables contaminated with *C. difficile*. Twelve of 18 recovered food isolates were toxigenic (toxinotypes 0, III, V, XII). The isolates belonged to 13 different PCR ribotypes, 001 being most common (5 isolates). Several food types with an increased potential of being contaminated with *C. difficile* were detected by surveillance.

**Conclusion:**

The three-year *C. difficile* testing within the national food surveillance revealed a low proportion of *C. difficile*-contaminated food and high genotype variability. Because the risk of *C. difficile* infection associated with *C. difficile-*contaminated food is unknown, no measures were recommended in the case of positive results.

## Introduction

*Clostridioides difficile* is an important cause of intestinal infections in humans. The global rates of *C. difficile* infections (CDI) have increased considerably over the past two decades [1-4]. In Slovenia, the reporting of CDI is obligatory. According to the report on infectious disease surveillance in Slovenia for the year 2017, provided by the National Institute for Public Health, the number of cases increased from 316 in the year 2013 to 665 in the year 2017 [5]. No community outbreaks were reported in Slovenia during this time and only a few outbreaks were reported in hospitals [6]. The reason for this increase is not completely clear, but could include improved reporting and changes in laboratory practice.

For more than a decade, the zoonotic potential and food-borne transmission of *C. difficile* have been hypothesised to contribute to its spread [7-11]. The zoonotic transmission is supported by the occurrence of *C. difficile* in animals, and the shared PCR ribotypes between humans and animals. A more precise whole-genome analysis has confirmed an overlap of genetically related strains between humans and animals [12-15].

Food studies have mainly focused on meat and meat products; however, raw and ready-to-eat vegetables, seafood and milk can also contain *C. difficile* [10,11,16]. Most of the reported prevalence rates vary from not detected to 12.5% in meat and 7.5% in vegetables [10,11,17], but can rise above 50.0% in seafood and root vegetables [18-21]. As with animal strains, PCR ribotypes of food isolates can also overlap with human PCR ribotypes and can be genetically undistinguishable from human isolates by MLVA, a method with higher discriminatory power than PCR ribotyping [16].

Because of increasing indications of moderate to high rates of *C. difficile* contamination of certain food, in 2015, the Administration of the Republic of Slovenia for Food Safety, Veterinary Sector and Plant Protection (Uprava za varno hrano, veterinarstvo in varstvo rastlin (UVHVVR)) included *C. difficile* in the national surveillance programme of raw meats. In 2016, the *C. difficile* surveillance was expanded to selected vegetables. The objective was to determine the burden of *C. difficile* in retail food in Slovenia based on systematic data collection and analysis. Here we present the results of *C. difficile* detection in Slovenian retail food based on 3 years of national food surveillance.

## Methods

### Participating laboratories

Two different institutions provide the sampling and microbiological analysis of food samples in national food surveillance program. Sampling and analysis of meat and seafood is performed by the National Veterinary Institute, which operates under the auspices of the Veterinary Faculty, University of Ljubljana. Food of non-animal origin is sampled and analysed by the National Laboratory for Health, Environment and Food (NLZOH). Both institutions followed the annual sampling instructions released by the UVHVVR. Results are reported to UVHVVR at the end of yearly surveillance and included in its annual report.

### Sampling

Different types of raw meat, including bovine or/and pork minced meat and meat preparations, poultry meat and poultry meat preparations were collected at food markets and grocery stores in all Slovenian regions from April to December 2015 and from March to December in both 2016 and 2017. Meat preparations are defined in European Union regulation (EC) 853/2004 as fresh meat, including meat that has been reduced to fragments, which has had foodstuffs, seasonings or additives added to it or which has undergone processes insufficient to modify the internal muscle fibre structure of the meat and thus to eliminate the characteristics of fresh meat [22]. Seafood was only sampled from March to December 2017 (excluding October).

Like retail meat samples, vegetables were sampled systematically in all Slovenian regions, but only from March to December in 2016 and 2017 (excluding April 2016 and November 2017). Samples were obtained from various retailers, including restaurants, food markets, grocery stores and supermarkets.

Information on sampling location and date was recorded for each collected sample.

A total of 336 samples of meat (raw and minced), 319 meat preparations and 17 seafood were tested between 2015 and 2017, with 4 to 24 samples tested per month. Samples were categorised as raw poultry meat (n = 60), meat preparations from poultry (n = 120), minced pork and/or beef meat (n = 60), meat preparations from pork and/or beef meat (n = 79), shrimps (n = 9) and bivalve molluscs (n = 8). (Table 1).

**Table 1:** Overview of the national food surveillance sampling plan and the number of samples tested for *Clostridioides difficile*, Slovenia, 2015–2017

Food type	Description	2015		2016		2017	
		All tested samples (n)	Samples tested for *C. difficile* (n)	All tested samples (n)	Samples tested for *C. difficile* (n)	All tested samples (n)	Samples tested for *C. difficile* (n)
**Animal origin**							
All types		345	119	363	130	462	87
Raw beef or/and pork	Minced meat and meat preparations	60	59	60	60	60	20
Raw poultry meat	Raw poultry meat and meat preparations, ducks	60	60	70	70	110	50
Seafood	Live bivalve molluscs (mussels, clams) and cooked crustaceans	10	0	21	0	22	17
Ready-to-eat meat products	Salami and sausages (fermented, dried, smoked), minced lard, other meat products	70	0	60	0	50	0
Milk and milk products	Raw milk and cheese (bovine, ovine, goat)	90	0	85	0	85	0
Fish and fish products	Fish pate; smoked, canned and raw fish	55	0	67	0	35	0
Eggs		0	0	0	0	100	0
**Non-animal origin** ^a^							
All types		335	NA	335	48	355	50
Vegetables	Ready-to-eat salads (from mixed vegetables, leaf salads, tomatoes, cucumbers)	70	NA	70	28	60	30
	Raw vegetables (leaf salads, root vegetables)	0	NA	20	20	20	20
	Sprouts, sprout seeds	15	NA	20	0	15	0
Fruit	Ready-to-eat fruit	10	NA	20	0	20	0
	Berries	10	NA	10	0	10	0
Nuts		20	NA	0	0	40	0
Ice cream		30	NA	50	0	20	0
Herbs, spices		10	NA	25	0	30	0
Ready-to-eat food	Confectionery	60	NA	40	0	40	0
	Sandwiches	40	NA	40	0	30	0
	Other ready-to-eat food	70	NA	40	0	70	0

NA: not applicable.

^a^ The national *C. difficile* surveillance programme of vegetables started in 2016.

A total of 98 vegetable samples were collected, with one to 13 samples tested per month. Samples included different types of raw green leafy salads (n = 21; green lettuce (n = 20) and lamb’s lettuce (n = 1)), ready-to-eat salads (n = 57) and root vegetables (n = 20; carrots, parsley, beetroot). Ready-to-eat salads were those made of leafy green lettuce (n = 41; green lettuce, radicchio, cabbage, rucola), cut cucumbers (n = 3), cut tomatoes (n = 7) or ready-to-eat salad combinations of two or more different kinds of fresh produce (n = 6; green lettuce, cabbage, spinach, tomatoes, cucumbers, beans, corn, carrots, onions) (Table 1).

### Origins of samples

*C. difficile* was found in our previous study on imported retail potatoes, indicating a potential way for *C. difficile* to spread between countries [21]. For this reason, information of food origin was also included in the analysis of the samples from the national food surveillance.

Of 319 meat and meat preparation samples, 266 were labelled ‘made in Slovenia’ (70.8%), 21 ‘made in Austria’ (6.6%), 11 ‘made in Croatia’ (3.4%), nine ‘made in Germany’ (2.8%), one ‘made in Hungary” (0.3%) and one ’made in Denmark’ (0.3%). A further five samples included ingredients from more than one country while another five had no information on origin available. Samples of shrimps (n = 9) originated from Lithuania, Denmark, Ecuador and the Czech Republic, while all samples of bivalve molluscs (n = 8) originated from Slovenia.

Of the 98 vegetable products, most (n = 81; 82.7%) were labelled ‘made in Slovenia’. Sixteen were made in Italy (n = 8) or other European countries (Austria (n = 1), Croatia (n = 1), Hungary (n = 1), Germany (n = 1), the Netherlands (n = 1), Poland (n = 2), Spain (n = 1)). No information on processing or origin was available for one sample.

### Isolation of *Clostridioides difficile*

Retail meat samples and seafood were analysed by the microbiological laboratory of the Institute of Food Safety, Feed and Environment, which is a part of the National Veterinary Institute. The isolation protocol for meat samples was previously established and evaluated by Biasizzo et al., 2018 [23], where samples spiked with 1,000, 100 and 10 CFU per sample were tested and 100% sensitivity was determined for all spiked samples.

Cycloserine cefoxitin fructose broth (90 ml) supplemented with taurocholic acid and lysozyme (TCCFB + L; prepared according to Biasizzo et al.) was added to 10 g of each meat sample in a sterile plastic blender bag and homogenised in a peristaltic blender (BagMixer, Interscience, St Nom la Bretèche, France) for 1 min. The samples were then incubated under anaerobic conditions at 37 °C for 7 days. The enriched samples were tested for *C. difficile* by quantitative real-time PCR and plated after spore selection by ethanol treatment onto blood agar plates (blood agar supplemented with 5% defibrinated bovine blood), *C. difficile* agar base with added *C. difficile* selective supplement and 7% (v/v) defibrinated horse blood ( cycloserine cefoxitin fructose agar (CCFA); all from Oxoid, Hampshire, United Kingdom (UK)) as previously described [23] and onto chromID *C. difficile* agar (bioMerieux, Lyon, France).

Retail vegetables were tested by NLZOH. Root and leafy vegetables were prepared differently, but for both, 25 g of the sample was analysed and the cultivation method was the same. Isolation was based on previously described cultivation method of Tkalec et al., 2019 [21] with detection threshold for root and leafy vegetables between 1 and 10 CFU per tested sample.

The recovery of *C. difficile* from root vegetables was assessed using sterile sponges (Polywipe, Medical Wire and Equipment (MWE), Corsham, UK), enrichment in BHIST broth (BHI supplemented with sodium taurocholate, yeast extract, L-cysteine, cycloserine and cefoxitin), spore selection by ethanol treatment and plating onto *C. difficile* selective agar (chromID *C. difficile* agar (bioMerieux)) [21]. Sampling with sponges is considerably more sensitive than using swabs [24] and can, together with enrichment step in cultivation, enable detection of 1 to 10 spores for swabbed potatoes [21].

The leafy vegetables were homogenised and incubated in blender bags (BagMixer, Interscience) in 200 ml of BHIST. *C. difficile* was isolated after the spore selection and plating onto chromogenic agar plates (bioMerieux) as described for root vegetables.

Identification of all presumptive *C. difficile* colonies was confirmed by mass spectrometry (MALDI-TOF Biotyper System, Bruker Daltonik, Bremen, Germany).

### Molecular characterisation of *Clostridioides difficile* isolates

All isolates were characterised by toxinotyping and PCR ribotyping. Toxinotyping was done by PCR amplification and restriction analysis of A3 and B1 fragments of *tcd*A and *tcd*B genes as previously described by Rupnik and Janezic [25]. The binary toxin gene was detected by partial amplification of *cdt*B [25].

PCR ribotyping was performed with Janezic primers as described by Janezic [26]. Profiles obtained by agarose-based PCR ribotyping were analysed by BioNumerics software version 7.6 (Applied Maths NV, Sint-Martens-Latem, Belgium) and compared with PCR ribotypes in our large PCR ribotype library that includes ca. 300 different PCR ribotypes, comprising isolates from Slovenia and abroad. Some of the reference library strains are representatives of internationally recognised ECDC/Leeds PCR ribotypes. If there was no match with these reference strains, the ribotype has a local designation (SLO+number).

### Statistical analysis

The data was analysed by R version 3.4.4 (R Foundation, Vienna, Austria). The analysis was performed for each categorical variable where the proportions were given. Proportions were compared using Fisher’s exact test. Statistical significance was held at p < 0.05.

## Results

In years 2015, 2016 and 2017, a total of 345, 363 and 462 food samples of animal origin were tested, respectively (Table 1). Only a proportion of the samples was included in *C. difficile* testing: 119 (34.7%) in 2015, 130 (35%) in 2016 and 87 (18.8%) in 2017. For food samples of non-animal origin, a total of 335 samples were tested in 2016 and 355 in 2017. Of those only a proportion of vegetables was tested for *C. difficile*: 48 (48.0%) in 2016 and 50 (62.5%) in 2017 (Table 1).This means that a total of 434 retail food samples collected as part of the national surveillance programme were tested for *C. difficile*. The overall prevalence of *C. difficile* in retail meat was 3.6% (12/336) (Table 2) and in retail vegetables it was 6.1% (6/98) (Table 3).

**Table 2:** Number and types of meat samples testing positive for *Clostridioides difficile* as a part of national food surveillance programme, Slovenia, 2015–2017

Year of sampling		2015						2016						2017						Total			
Type of meat^a^		Samples tested (n)	Positive samples (n)	Percentage positive (%)	95% CI (%)	PCR ribotype	Toxinotype (Binary toxin *cdt*B gene)	Samples tested (n)	Positive samples (n)	Percentage positive (%)	95% CI (%)	PCR ribotype	Toxinotype (Binary toxin *cdt*B gene)	Samples tested (n)	Positive samples (n)	Percentage positive (%)	95% CI (%)	PCR ribotype	Toxinotype (Binary toxin *cdt*B gene)	Total tested (n)	Total positive (n)	Percentage positive (%)	95 CI (%)
Beef, pork	Minced meat	30	0	0.0	NA	NA	NA	30	0	0.0	NA	NA	NA	0	NA	NA	NA	NA	NA	**60**	**0**	**0.0**	**NA**
	Meat preparations^b^	29	1	3.4	0.0–10.0	078	V (BTb+)	30	1	3.3	0.0–9.7	SLO 283	Tox- (BTb-)	20	1	5.0	0.0–14.6	SLO 057	Tox- (BTb-)	**79**	**3**	**3.8**	**0.0–8.0**
Poultry	Meat	30	2	6.7	0.0–15.6	014/020 015	0 (BTb-) IX (BTb+)	30	1	3.3	0.0–9.7	001	0 (BTb-)	0	NA	NA	NA	NA	NA	**60**	**3**	**5.0**	**0.0–10.5**
	Meat preparations^b^	30	0	0.0	NA	NA	NA	40	3	7.5	0.0–15.7	SLO 052 001^c^	0 (BTb-) 0 (BTb-)	50	2	4.0	0.0–9.4	001 087	0 (BTb-) 0 (BTb-)	**120**	**5**	**4.2**	**0.6–7.8**
Seafood	Bivalve molluscs, shrimps	0	NA	NA	NA	NA	NA	0	NA	NA	NA	NA	NA	17	1	5.9	0.0–17.1	010	Tox- (BTb-)	**17**	**1**	**5.9**	**0–7.1**
**Total**		**119**	**3**	**2.5**	**0.0–5.3**	**3**	**3**	**130**	**5**	**3.8**	**0.5–7.1**	**3**	**2**	**87**	**4**	**4.6**	**0.2–9.0**	**4**	**2**	**336**	**12**	**3.6**	**1.6–5.6**

BTb-: binary toxin gene absent; BTb+: binary toxin gene present; CI: confidence interval; NA: not applicable; Tox-: non-toxigenic strain.

^a^ All tested meat and meat products were raw.

^b^ Meat preparations refer to raw meat with additional ingredients.

^c^ PCR ribotype 001 was detected in two samples.

**Table 3:** Number and types of vegetables testing positive for *Clostridioides difficile* as a part of national food surveillance programme, Slovenia, 2016–2017

Year of sampling		2016						2017						Total			
Type of vegetable		Tested samples (n)	Positive samples (n)	Percentage positive (%)	95% CI (%)	PCR ribotype	Toxinotype^a^ (Binary toxin *cdt*B gene)	Tested samples (n)	Positive samples (n)	Percentage positive (%)	95% CI (%)	PCR ribotype	Toxinotype^a^ (Binary toxin *cdt*B gene)	Total tested (n)	Total positive (n)	Percentage positive (%)	95% CI (%)
Raw	Leaf salads	1	1	100	NA	SLO 248	Tox- (BTb-)	20	2	10.0	0.0–23.1	001 012	0 (BTb-) 0 (BTb-)	**21**	**3**	**14.3**	**0.0–29.3**
	Root vegetables (carrot, parsley, beetroot)	20	1	5.0	0.0–14.6	SLO 128	III (BTb+)	0	NA	NA	NA	NA	NA	**20**	**1**	**5.0**	**0.0–14.6**
Ready-to-eat salads	Leaf salads	21	0	0	NA	NA	NA	20	2	10.0	0.0–23.1	010 SLO 028	Tox- (BTb-) Tox- (BTb-)	**41**	**2**	**4.9**	**0.0–11.5**
	Tomatoes, cucumbers	3	0	0	NA	NA	NA	8	0	0	NA	NA	NA	**11**	**0**	**0.0**	**NA**
	Salads from mixed vegetables (green salad, tomato, carrot, corn, beans, onion)	3	0	0	NA	NA	NA	2	0	0	NA	NA	NA	**5**	**0**	**0.0**	**NA**
**Total**		**48**	**2**	**4.2**	**0.0–9.9**	**2**	**2**	**50**	**4**	**8.0**	**0.5–15.5**	**4**	**2**	**98**	**6**	**6.1**	**1.4–10.8**

BTb-: binary toxin gene absent; BTb+: binary toxin gene present; CI: confidence interval; NA: not applicable; Tox-: non-toxigenic strain.

^a^ The toxinotyping results were congruent with toxin testing results with rapid membrane enzyme immunoassay C. DIFF QUIK CHEK COMPLETE (Alere, Galway, Ireland) for all the isolates.

### *Clostridioides difficile* in retail meat and seafood

In the year 2015, three of 119 meat samples (2.5%) tested positive for *C. difficile*, five of 130 meat samples (3.9%) in 2016 and four of 87 meat/seafood samples (4.6%) tested positive for *C. difficile* in 2017 (Table 2). Overall, *C. difficile* was isolated from 12 of 336 meat samples; five poultry meat preparations, three beef and/or pork meat preparations, three raw poultry meat and one bivalve molluscs. *C. difficile* was not detected in any of the 56 tested pork and/or beef minced meat samples. Of the 12 *C. difficile-*positive samples, 10 were made in Slovenia (3.8% of all tested samples made in Slovenia) and two were made in Austria (9.5% of all tested samples made in Austria).

There was no significant statistical difference in *C. difficile* prevalence between different meat types/categories or sampling locations (food markets vs grocery stores) (p > 0.05).

### *Clostridium difficile* in retail fresh vegetables

In 2016, two of 48 tested samples were *C. difficile-*positive and four of 50 samples tested positive in the year 2017 (Table 3). Collectively, *C. difficile* was isolated from six of 98 (6.1%) tested samples, in particular from three of 21 raw leaf salads, one of 20 root vegetables and two of 57ready-to-eat salads (Table 3). Comparison of *C. difficile* prevalence according to sample type (root, unprocessed salad, ready-to-eat salad) and location of sampling (supermarket, food market, farm, catering/restaurants) showed no significant statistical difference between the samples (p > 0.05).

Vegetables from three of six *C. difficile*-positive samples were grown in Italy, two in Slovenia and one in Poland. None of the positive vegetables shared sampling time point or location of sampling.

### PCR ribotypes and toxinotypes in retail food samples

The 18 *C. difficile* isolates obtained from food belonged to 13 different PCR ribotypes (Tables 2 and 3). The most prevalent PCR ribotype was PCR ribotype 001 (n = 5), followed by 010 (n = 2). Both were detected in food of animal and non-animal origin. The other 11 PCR ribotypes were detected only once. One nontoxigenic PCR ribotype, SLO 283, was new to our PCR ribotype library. It was isolated from a beef/pork meat preparation of Slovenian origin.

Two thirds of the isolates were toxigenic (n = 12). Nine belonged to toxinotype 0 (BTb-) and the remaining three to toxinotypes III (BTb+), V (BTb+) and IX (BTb+) (Tables 2 and 3). One third of all isolates were non-toxigenic (n = 6), comprising three of six vegetable isolates (Table 3), two of 11 (meat isolates, as well as the isolate recovered from seafood (Table 2).

The majority of PCR ribotypes were already found in Slovenia, either in patients, animals, food or the environment (Table 4).

**Table 4:** Comparison of *Clostridioides. difficile* PCR ribotypes detected in the national food surveillance to strains isolated in Slovenia from different reservoirs^a^, 2008–2017

PCR ribotype^b^	Total number of strains	Reservoir			
		Human	Animal	Environment	Food
001	217	187	16	14	7
010	187	62	22	103	0
012	69	58	2	9	2
014/020	826	556	72	198	17
015	46	36	1	9	0
078	45	28	5	12	0
087	36	36	0	0	0
SLO 028	5	4	0	1	0
SLO 057	35	6	13	16	2
SLO 128	1	1	0	0	0
SLO 248	3	1	0	2	0

^a^ The strains presented in the table were isolated from 2008 to the end of the current surveillance and are part of our strain collection (data not shown).

^b^ Two PCR ribotypes are not included in the table: SLO 283 was unique to our strain collection while for SLO 052, a single strain was present in the collection and originated from Croatia.

## Discussion

*C. difficile* can be found in different types of food and the reported contamination rates vary between food types, countries and studies. The main novelty of this report is that it describes the first documented long-term, national *C. difficile* food surveillance performed thus far.

We found that the proportion of *C. difficile* positive meat and vegetable samples numerically increased every year. However, the number of tested samples was too low to speculate on any possible trends. The changes in samples sizes and produce types could have contributed to this observation. Except for vegetables in 2017, the average annual positivity rate for food of animal or non-animal source was 4.6% or lower.

The proportion of meat samples positive for *C. difficile* detected during Slovenian food surveillance varied from 2.5% to 4.6% annually, and was 3.6% for entire 3-year interval. There are no published data available for Slovenia on meat contamination with *C. difficile*. In previous small sporadic pilot studies performed by NLZOH where 124 meat samples of pork, beef, sheep and poultry were tested, all were negative for *C. difficile* (Tkalec and Rupnik; data not shown). Therefore, it seems that the systematic sampling and optimised methodology [23] used in national surveillance contributed to better detection. Early studies about possible presence of *C. difficile* in food of animal origin from North America reported high contamination rates of meat and meat products (20% in beef and veal ground meat in Canada [27]; 14.3% to 62.5% depending on the meat sample type, in the US [28]). These high contamination rates have not been reported in subsequent European or other non-European studies [8,10,11,17]. In general, the meat testing results were comparable to other European countries reporting from none to 6.3% [10,11,17] and to most of the recent reports from non-European countries reporting from none to 12.5% contamination rate in different types of meat [10,17,29]. In our sample set, beef/pork minced meat were always negative for *C. difficile;* this was also the case in some reports from Switzerland and Austria [30,31]. However, other studies from Austria, Belgium, France and Sweden reported positivity rates from 1.9% to 4.7% [11,17]. Poultry meat or meat preparations are not often included in the testing. In contrast to our finding of overall 4.4% (8/180) of *C. difficile* positive poultry samples, a 2.7% positivity rate in poultry meat was reported from the Netherlands while *C. difficile* was not detected in any poultry meat sample in Austria or Sweden [30,32].

The contamination rate of bivalve molluscs (5.9%; n = 1) was determined on a small number of samples (n = 17). But similar percentages (3.9%, 4.5% and 4.8%) were reported for Italy, Texas and Canada, respectively [17,29]. It seems that presence of *C. difficile* in mussels varies widely, as two other Italian studies found 49% (26 of 53 samples, and 75% (four of six samples) of mussels collected at farms or retailers in Naples, Italy to be positive for *C. difficile* [18,19].

The proportion of *C. difficile*-positive vegetable samples (6.1%) is comparable to the low to moderate percentages of contaminated vegetables and ready-to-eat salads reported by others [10,33-35]. Again, such comparisons are difficult not only because of the difference in methodology, but also because of heterogeneity of sampled vegetables. For this reason, the proportion of *C. difficile*-positive vegetable samples found during national *C. difficile* food surveillance cannot be directly compared with our previous study (9.4%) [21]. Leaf salads were relatively often contaminated with *C. difficile* in Slovenian food surveillance (14.3% of 21 samples). There is not much data on the testing of raw leaf salads, but a previous Slovenian survey found 8.8% of retail leaf salads positive for *C. difficile* [21], while a report from Iran found 5.7% positive and two studies from the US found 4.6% and 13.8% positive, respectively [29]. Ready-to-eat salads have been tested in France and Scotland, with 2.9 and 7.3% of samples positive, respectively [33,36]. However, the type of the ready-to-eat salads is vaguely described in most studies.

Of the root vegetables we tested (carrot, parsley and beetroot), only a single parsley sample was contaminated with *C. difficile*. An Australian study that tested organic root vegetables reported much higher positivity rates; 22.2% for beetroot, 5.6% for onions and 5.3% for carrots [20]. The vegetable reported by us and others to have a very high contamination rate (from 28.0% to up to 55.6%) is the potato [20,21], but potatoes were not included in the Slovenian food surveillance programme as these data were not available when the programme was started.

A large variety of PCR ribotypes and toxinotypes was found and it was slightly higher in vegetables than in meat. The six strains isolated from vegetables belonged to six different PCR ribotypes while the 12 strains isolated from meat belonged to nine different PCR ribotypes. Only ribotypes 001 and 010 were shared between meat/seafood and vegetables. All the strains of PCR ribotype 001 originated from Slovenian samples, while samples yielding strains of PCR ribotype 010 were from Italy (salad) and Slovenia (meat). Both PCR ribotypes 001 and 010 are rather diverse, and whole genome sequencing should be used to confirm the genetic (non)clonality. Of the 13 detected PCR ribotypes, only one was new to our PCR library. Some of the detected PCR ribotypes, such as 001, 010 and 078, are among the most prevalent in different human, animal and environmental studies from several countries, suggesting that certain PCR ribotypes are well adapted to survival in several different niches.

Some *C. difficile*-positive food samples in the food surveillance were of non-Slovenian origin. Another group of positive samples included foodstuffs that were composed of several ingredients, some of which had non-Slovenian origin. Positive samples or only their ingredients originated from Italy, Poland, Austria, Belgium and Lithuania. Therefore, as suggested earlier by the authors [21], food in general could possibly contribute to cross-border transmissions.

Theoretically, the *C. difficile* contamination sources of food could be very heterogeneous. For meat, the most direct way is faecal contamination during slaughtering, but post-production processing could also play a role. Despite a very low, close to zero, reported prevalence of *C. difficile* in poultry before slaughter by some groups [8,37], higher contamination rates, up to 12.8%, of raw poultry meat have been documented in North America [37,38] and in our surveillance (5.0%). This suggests a post-slaughter contamination source in the production line. A possible source for the contamination of both meat and vegetables could also be via contaminated hands during handling. The main sources of vegetable contamination are soil or indirect faecal contamination via irrigation or manuring [39]. Non-toxigenic strains are often found in rural soils [40] and were also commonly isolated from vegetables during our surveillance. This constant environmental exposure of vegetables to *C. difficile* spores is probably one of the reasons for higher contamination rates of vegetables compared to meat and meat products.

*C. difficile* food surveillance had several limitations. One of the limitations is that no baseline contamination rates were known for Slovenia. It is for this reason that testing for *C. difficile* was initially added for all samples of raw meat, meat preparations and raw vegetable planned to be sampled for other microbiological parameters in the surveillance programme. Another limitation is the difference in the methodology used by both institutions providing the testing. Institutions used their own optimised methods that differed in the quantity of the sample (higher for vegetables), enrichment medium and use of molecular methods for screening of positive enrichment cultures, used for meat only. This difference in the methodology could have contributed to the higher proportion of vegetable samples positive for *C. difficile* (6.1%) compared with meat samples (3.6%). However, both methods were previously shown to have detection limits of at least 10 spores per sample. Furthermore, because of different sampling procedures, different types of tested food, bias because of the low sample number and, more notably, the differences in detection methods [11], comparisons among published studies should be made cautiously.

One of the aims of the national food surveillance is consumer protection. However, the detection of *C. difficile* in food during the surveillance has not resulted in any recommended measures because the risk associated with contaminated *C. difficile* food is unknown. Given the low detected prevalence of *C. difficile* contaminated food samples, the food safety risks for the tested food types are likely very low. On the other hand, although the levels of food contamination with *C. difficile* spores are usually low [41], the constant exposure to the low spore levels in combination with a disrupted gut microbiota or immune incompetence could represent increased risk for *C. difficile* infection (CDI).

In summary, *C. difficile* testing within the national food surveillance programme over the course of 3 years revealed a low percentage of *C. difficile-*contaminated food and a high genotype variability. Surveillance detected that some food types included in regular food surveillance, such as meat preparations and leafy salads, are more likely to be contaminated. Because the risk associated with *C. difficile* contaminated food is unknown, no measures have been recommended in terms of any of the 18 foods positive for *C. difficile*.

## References

[1] Lessa FC Winston LG McDonald LC Emerging Infections Program C. difficile Surveillance Team Burden of Clostridium difficile infection in the United States. N Engl J Med. 2015;372(24):2369-70. 26061850 10.1056/NEJMc1505190 PMC10880113

[2] van Dorp SM Kinross P Gastmeier P Behnke M Kola A Delmée M Standardised surveillance of Clostridium difficile infection in European acute care hospitals: a pilot study, 2013. Euro Surveill. 2016;21(29):30293. 10.2807/1560-7917.ES.2016.21.29.30293 27472820

[3] Kola A Wiuff C Akerlund T van Benthem BH Coignard B Lyytikäinen O Survey of Clostridium difficile infection surveillance systems in Europe, 2011. Euro Surveill. 2016;21(29):30291. 10.2807/1560-7917.ES.2016.21.29.30291 27469420

[4] McFarland LV, Surawicz CM, Vindigni SM. Clostridium difficile infections: Epidemiology, diagnosis, and treatment. Evidence-Based Gastroenterology and Hepatology. 2019;4e:284-305.

[5] Nacionalni inštitut za javno zdravje (NIJZ). [National Institute for Public Health]. Epidemiološko spremljanje nalezljivih bolezni v Sloveniji v letu 2017. [Report on infectious disease surveillance in Slovenia for the year 2017]. Ljubljana: NIJZ; 2018. Slovenian. Available from: https://www.nijz.si/sites/www.nijz.si/files/uploaded/epidemiolosko_spremljanje_nb_v_sloveniji_2017_november2018_1.pdf

[6] Nacionalni inštitut za javno zdravje (NIJZ). [National Institute for Public Health]. Epidemiološko spremljanje nalezljivih bolezni v Sloveniji v letu 2015. [Report on infectious disease surveillance in Slovenia for the year 2015]. Ljubljana: NIJZ; 2016. Slovenian. Available from: http://www.nijz.si/sites/www.nijz.si/files/datoteke/epidemiolosko_spremljanje_nb_v_letu_2015.pdf

[7] Rupnik M Is Clostridium difficile-associated infection a potentially zoonotic and foodborne disease? Clin Microbiol Infect. 2007;13(5):457-9. 10.1111/j.1469-0691.2007.01687.x 17331126

[8] Rodriguez-Palacios A Borgmann S Kline TR LeJeune JT Clostridium difficile in foods and animals: history and measures to reduce exposure. Anim Health Res Rev. 2013;14(1):11-29. 10.1017/S1466252312000229 23324529

[9] Brown AWW Wilson RB *Clostridium difficile* colitis and zoonotic origins-a narrative review. Gastroenterol Rep (Oxf). 2018;6(3):157-66. 10.1093/gastro/goy016 30151199 PMC6101521

[10] Rodriguez Diaz C Seyboldt C Rupnik M Non-human C. difficile reservoirs and sources: animals, food, environment. Adv Exp Med Biol. 2018;1050:227-43. 10.1007/978-3-319-72799-8_13 29383672

[11] Candel-Pérez C Ros-Berruezo G Martínez-Graciá C A review of Clostridioides [Clostridium] difficile occurrence through the food chain. Food Microbiol. 2019;77:118-29. 10.1016/j.fm.2018.08.012 30297042

[12] Knetsch CW Connor TR Mutreja A van Dorp SM Sanders IM Browne HP Whole genome sequencing reveals potential spread of Clostridium difficile between humans and farm animals in the Netherlands, 2002 to 2011. Euro Surveill. 2014;19(45):20954. 10.2807/1560-7917.ES2014.19.45.20954 25411691 PMC4518193

[13] Knight DR Squire MM Collins DA Riley TV Genome analysis of Clostridium difficile PCR Ribotype 014 Lineage in Australian pigs and humans reveals a diverse genetic repertoire and signatures of long-range interspecies transmission. Front Microbiol. 2017;7:2138. 10.3389/fmicb.2016.02138 28123380 PMC5225093

[14] Knetsch CW Kumar N Forster SC Connor TR Browne HP Harmanus C Zoonotic transfer of Clostridium difficile harboring antimicrobial resistance between farm animals and humans. J Clin Microbiol. 2018;56(3):e01384-17. 29237792 10.1128/JCM.01384-17 PMC5824051

[15] Knight DR Riley TV Genomic delineation of zoonotic origins of Clostridium difficile. Front Public Health. 2019;7:164. 10.3389/fpubh.2019.00164 31281807 PMC6595230

[16] Romano V Pasquale V Lemee L El Meouche I Pestel-Caron M Capuano F Clostridioides difficile in the environment, food, animals and humans in southern Italy: Occurrence and genetic relatedness. Comp Immunol Microbiol Infect Dis. 2018;59:41-6. 10.1016/j.cimid.2018.08.006 30290886

[17] Rodriguez C Taminiau B Van Broeck J Delmée M Daube G Clostridium difficile in food and animals: A comprehensive review. Adv Exp Med Biol. 2016;932:65-92. 10.1007/5584_2016_27 27350639

[18] Pasquale V Romano VJ Rupnik M Dumontet S Cižnár I Aliberti F Isolation and characterization of Clostridium difficile from shellfish and marine environments. Folia Microbiol (Praha). 2011;56(5):431-7. 10.1007/s12223-011-0068-3 21901293

[19] Pasquale V Romano V Rupnik M Capuano F Bove D Aliberti F Occurrence of toxigenic Clostridium difficile in edible bivalve molluscs. Food Microbiol. 2012;31(2):309-12. 10.1016/j.fm.2012.03.001 22608238

[20] Lim SC Foster NF Elliott B Riley TV High prevalence of Clostridium difficile on retail root vegetables, Western Australia. J Appl Microbiol. 2018;124(2):585-90. 10.1111/jam.13653 29193458

[21] Tkalec V Janezic S Skok B Simonic T Mesaric S Vrabic T High Clostridium difficile contamination rates of domestic and imported potatoes compared to some other vegetables in Slovenia. Food Microbiol. 2019;78:194-200. 10.1016/j.fm.2018.10.017 30497603

[22] European Commission. Regulation (EC) No 853/2004 of the European Parliament and of the Council of 29 April 2004 laying down specific hygiene rules for food of animal origin. 30.4.2004: L 139. [Accessed 17 Apr 2020]. Available from: http://data.europa.eu/eli/reg/2004/853/2014-06-01

[23] Biasizzo M Vadnjal S Henigman U Krizman M Kirbis A Jamnikar-Ciglenecki U Development and validation of a new protocol for detecting and recovering Clostridium difficile from meat samples. J Food Prot. 2018;81(4):561-8. 10.4315/0362-028X.JFP-17-354 29517350

[24] Best EL Fawley WN Parnell P Wilcox MH The potential for airborne dispersal of Clostridium difficile from symptomatic patients. Clin Infect Dis. 2010;50(11):1450-7. 10.1086/652648 20415567

[25] Rupnik M Clostridium difficile toxinotyping. Methods Mol Biol. 2010;646:67-76. 10.1007/978-1-60327-365-7_5 20597003

[26] Janezic S Direct PCR-Ribotyping of Clostridium difficile. Methods Mol Biol. 2016;1476:15-21. 10.1007/978-1-4939-6361-4_2 27507330

[27] Rodriguez-Palacios A Staempfli HR Duffield T Weese JS Clostridium difficile in retail ground meat, Canada. Emerg Infect Dis. 2007;13(3):485-7. 10.3201/eid1303.060988 17552108 PMC2725909

[28] Songer JG Trinh HT Killgore GE Thompson AD McDonald LC Limbago BM Clostridium difficile in retail meat products, USA, 2007. Emerg Infect Dis. 2009;15(5):819-21. 10.3201/eid1505.081071 19402980 PMC2687047

[29] Warriner K Xu C Habash M Sultan S Weese SJ Dissemination of Clostridium difficile in food and the environment: Significant sources of C. difficile community-acquired infection? J Appl Microbiol. 2017;122(3):542-53. 10.1111/jam.13338 27813268

[30] Indra A Lassnig H Baliko N Much P Fiedler A Huhulescu S Clostridium difficile: a new zoonotic agent? Wien Klin Wochenschr. 2009;121(3-4):91-5. 10.1007/s00508-008-1127-x 19280132

[31] Hoffer E Haechler H Frei R Stephan R Low occurrence of Clostridium difficile in fecal samples of healthy calves and pigs at slaughter and in minced meat in Switzerland. J Food Prot. 2010;73(5):973-5. 10.4315/0362-028X-73.5.973 20501051

[32] Von Abercron SM Karlsson F Wigh GT Wierup M Krovacek K Low occurrence of Clostridium difficile in retail ground meat in Sweden. J Food Prot. 2009;72(8):1732-4. 10.4315/0362-028X-72.8.1732 19722410

[33] Bakri MM Brown DJ Butcher JP Sutherland AD Clostridium difficile in ready-to-eat salads, Scotland. Emerg Infect Dis. 2009;15(5):817-8. 10.3201/eid1505.081186 19402979 PMC2687046

[34] Metcalf DS Costa MC Dew WM Weese JS Clostridium difficile in vegetables, Canada. Lett Appl Microbiol. 2010;51(5):600-2. 10.1111/j.1472-765X.2010.02933.x 21069911

[35] Eckert C Burghoffer B Barbut F Contamination of ready-to-eat raw vegetables with Clostridium difficile in France. J Med Microbiol. 2013;62(9):1435-8. 10.1099/jmm.0.056358-0 23449876

[36] Eckert C Burghoffer B Barbut F Contamination of ready-to-eat raw vegetables with Clostridium difficile in France. J Med Microbiol. 2013;62(9):1435-8. 10.1099/jmm.0.056358-0 23449876

[37] Harvey RB Norman KN Andrews K Hume ME Scanlan CM Callaway TR Clostridium difficile in poultry and poultry meat. Foodborne Pathog Dis. 2011;8(12):1321-3. 10.1089/fpd.2011.0936 21877928

[38] Weese JS Reid-Smith RJ Avery BP Rousseau J Detection and characterization of Clostridium difficile in retail chicken. Lett Appl Microbiol. 2010;50(4):362-5. 10.1111/j.1472-765X.2010.02802.x 20102510

[39] Atidégla SC Huat J Agbossou EK Saint-Macary H Glèlè Kakai R Vegetable Contamination by the Fecal Bacteria of Poultry Manure: Case Study of Gardening Sites in Southern Benin. Int J Food Sci. 2016;2016:4767453. 10.1155/2016/4767453 27069914 PMC4812396

[40] Janezic S Potocnik M Zidaric V Rupnik M Highly divergent Clostridium difficile strains isolated from the environment. PLoS One. 2016;11(11):e0167101. 10.1371/journal.pone.0167101 27880843 PMC5120845

[41] Weese JS Avery BP Rousseau J Reid-Smith RJ Detection and enumeration of Clostridium difficile spores in retail beef and pork. Appl Environ Microbiol. 2009;75(15):5009-11. 10.1128/AEM.00480-09 19525267 PMC2725490

